# Poster Session I - A162 COLON-SPECIFIC MINIMALLY INVASIVE ENDOSCOPIC RESECTION OUTCOMES: A PROSPECTIVE MULTICENTER COHORT OF 1000 LARGE NON-PEDUNCULATED COLONIC POLYPS

**DOI:** 10.1093/jcag/gwaf042.162

**Published:** 2026-02-13

**Authors:** A Karimuddin, S Sui, A Hu, S Pang, R Trasolini, D Motomura, E Lam, N C Shahidi

**Affiliations:** St Paul’s Hospital, Vancouver, BC, Canada; St Paul’s Hospital, Vancouver, BC, Canada; St Paul’s Hospital, Vancouver, BC, Canada; St Paul’s Hospital, Vancouver, BC, Canada; Vancouver General Hospital, Vancouver, BC, Canada; St Paul’s Hospital, Vancouver, BC, Canada; St Paul’s Hospital, Vancouver, BC, Canada; St Paul’s Hospital, Vancouver, BC, Canada

## Abstract

**Background:**

Endoscopic resection has revolutionized the management of large (≥ 20 mm) non-pedunculated colonic polyps (LNPCPs) with international data demonstrating its efficacy, safety and cost-effectiveness. Canadian outcome data from tertiary resection programs remains limited

**Aims:**

Evaluate the outcomes of endoscopic resection of LNPCPs in a Canadian tertiary referral program.

**Methods:**

Consecutive adult patients referred for the management of a LNPCP were enrolled in a prospective multi-centre cohort (ClinicalTrials.gov identifier: NCT05402696). Endoscopic modality selection (endoscopic mucosal resection [EMR], cold snare resection [CSR], or endoscopic submucosal dissection [ESD]) was at the discretion of the endoscopist. The primary outcome was technical success (removal of all polypoid tissue at index resection). Secondary outcomes included peri-procedural adverse events (intra-procedural perforation [IPP], clinically significant post-endoscopic resection bleeding [CSPEB], delayed perforation), and recurrence at first surveillance colonoscopy (SC1).

**Results:**

From 06/2022 – 10/2025, 1138 large colorectal lesions were enrolled; 38 did not undergo endoscopic resection and 97 were located in the rectum. Ultimately 1003 LNPCPs were included for analysis. Median patient age was 68 years (IQR 61-73 years), and 45.4% of patients were female. Median lesion size was 25 mm (IQR 20-35mm) with 46.9% located in the cecum/ascending colon. The predominant morphology was Paris 0-IIA (66.1%) with 44.8% demonstrating granular topography. On histopathology, 62.5% were adenomas and 30.6% were sessile serrated lesions. Overall cancer frequency was 3.4%. Predominant resection modalities included piecemeal EMR (55.4%), CSR (26.3%), and ESD (9.5%). Technical success was achieved in 98.3%. Intraprocedural perforation occurred in 3.0% and CSPEB occurred in 4.7% with no delayed perforations. Hospital admission was required in 7.5% of patients, primarily due to CSPEB. Recurrence at SC1 was identified in 2.3%.

**Conclusions:**

Our findings support that tertiary tissue resection programs demonstrate high performance in minimally invasive endoscopic resection for LNPCPs.

**Funding Agencies:**

None

